# Alpha-2-Macroglobulin, a Hypochlorite-Regulated Chaperone and Immune System Modulator

**DOI:** 10.1155/2019/5410657

**Published:** 2019-07-22

**Authors:** Jordan H. Cater, Mark R. Wilson, Amy R. Wyatt

**Affiliations:** ^1^Illawarra Health and Medical Research Institute and School of Chemistry and Molecular Bioscience, University of Wollongong, New South Wales 2522, Australia; ^2^College of Medicine and Public Health, Flinders University, South Australia 4052, Australia; ^3^Centre for Neuroscience, Flinders University, South Australia 4052, Australia

## Abstract

Alpha-macroglobulins are ancient proteins that include monomeric, dimeric, and tetrameric family members. In humans, and many other mammals, the predominant alpha-macroglobulin is alpha-2-macroglobulin (*α*_2_M), a tetrameric protein that is constitutively abundant in biological fluids (e.g., blood plasma, cerebral spinal fluid, synovial fluid, ocular fluid, and interstitial fluid). *α*_2_M is best known for its remarkable ability to inhibit a broad spectrum of proteases, but the full gamut of its activities affects diverse biological processes. For example, *α*_2_M can stabilise and facilitate the clearance of the Alzheimer's disease-associated amyloid beta (A*β*) peptide. Additionally, *α*_2_M can influence the signalling of cytokines and growth factors including neurotrophins. The results of several studies support the idea that the functions of *α*_2_M are uniquely regulated by hypochlorite, an oxidant that is generated during inflammation, which induces the native *α*_2_M tetramer to dissociate into dimers. This review will discuss the evidence for hypochlorite-induced regulation of *α*_2_M and the possible implications of this in neuroinflammation and neurodegeneration.

## 1. Structure and Function

**Figure 1:**
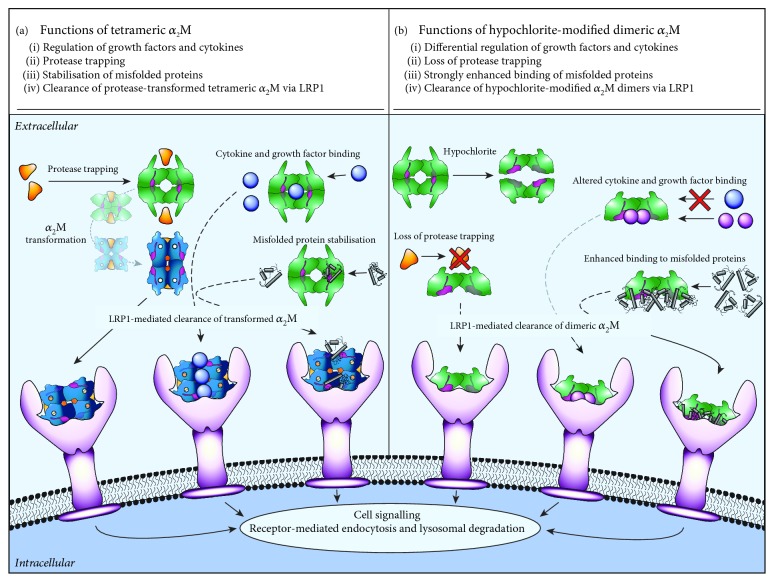
Schematic diagram showing the function consequences of hypochlorite-induced modification of *α*_2_M. (a) Native *α*_2_M, a tetramer (shown in green), is constitutively present in biological fluids and covalently binds to a broad range of proteases. Binding to proteases results in a conformational change that exposes the binding site on *α*_2_M for LRP1, which is responsible for the clearance of the protease-transformed *α*_2_M complex (shown in dark blue). *α*_2_M also binds to a large number of noncovalent ligands including cytokines and misfolded proteins. In many cases, noncovalent binding of ligands occurs preferentially to the protease-transformed conformation (not shown). In the instance that native *α*_2_M binds noncovalently to a nonprotease substrate, protease interaction is required to enable clearance of the complex via LRP1. (b) Reaction with hypochlorite induces the dissociation of the native *α*_2_M tetramer into dimers. This process abolishes the protease-trapping activity of *α*_2_M; however, the binding to some cytokines (i.e., TNF-*α*, IL-2, and IL-6) and misfolded proteins is enhanced. On the other hand, the binding of *α*_2_M to other noncovalent ligands (i.e., *β*-NGF, PDGF-BB, TGF-*β*1, and TGF-*β*2) is reduced. The dissociation of the native *α*_2_M tetramer into dimers reveals the binding site on *α*_2_M for LRP1. Therefore, *α*_2_M dimers can facilitate the clearance of substrates in a protease-independent manner. N.B.: Inflammatory processes potentially elevate levels of protease-transformed *α*_2_M and hypochlorite-modified *α*_2_M dimers, concomitantly.

*α*_2_M is a secreted protein that is present at 1.5–2 mg mL^−1^ and 1.0–3.6 *μ*g mL^−1^ in human blood plasma and cerebral spinal fluid, respectively [1, 2]. The cage-like structure of *α*_2_M (720 kDa) is formed by the assembly of four 180 kDa subunits into two disulfide-linked dimers, which noncovalently associate to complete the tetrameric quaternary structure of the protein [3]. A bait region that contains a large number of protease cleavage sites is responsible for the incredibly diverse range of proteases that interact with *α*_2_M [4]. Cleavage of the *α*_2_M bait region, which is in close physical proximity to a reactive thioester bond, results in covalent trapping of proteases within a steric cage [5]. This process involves a substantial conformational change that generates a compact tetrameric form [6] and reveals the binding site for the low-density lipoprotein receptor-related protein-1 (LRP1) [7, 8] (Figure 1(a)). For the purpose of this review, the compact tetrameric protease-bound form of *α*_2_M is referred to as transformed *α*_2_M. Transformed *α*_2_M (covalently bound to up to two protease molecules) is rapidly cleared from the circulation via LRP1-facilitated endocytosis (Figure 1(a)). As such, *α*_2_M can efficiently inhibit a myriad of extracellular processes that are dependent on proteolysis.

Consistent with having an ancient origin in innate immunity, *α*_2_M is a promiscuous protein that noncovalently binds to a diverse range of nonprotease ligands including cytokines [9, 10], growth factors [9–14], apolipoproteins [15], and misfolded proteins [16–20]. Many noncovalent ligands of *α*_2_M including the Alzheimer's disease-associated A*β* peptide [21], neurotrophins [14], and tumour necrosis factor-alpha (TNF-*α*) preferentially bind to transformed *α*_2_M which is generated following the reaction of native *α*_2_M with a protease or with small nucleophilic compounds that also target the *α*_2_M thioester bond [6]. In these cases, it is proposed that transformed *α*_2_M acts to limit the activities of noncovalently bound ligands by facilitating their disposal via LRP1 [10, 22] (Figure 1(a)). On the other hand, *α*_2_M can control signalling pathways via alternative mechanisms. For example, the binding of *α*_2_M to phosphorylated insulin-like growth factor binding protein-1 abrogates its inhibitory effects on insulin-like growth factor-1 (IGF-1); therefore, in some scenarios, *α*_2_M can potentiate growth factor signalling [13]. Another example whereby *α*_2_M is reported to potentiate growth factor signalling involves the pronerve growth factor (pro-NGF), which induces the expression of TNF-*α* via stimulating the neurotrophin receptor p75 [11]. Although *α*_2_M potentiates pro-NGF signalling *in vitro*, *α*_2_M is reported to inhibit the activity of mature NGF by binding either to NGF or to Trk receptors [12, 23, 24].

The accumulation of misfolded proteins is inherently deleterious to living organisms and underlies the pathology of many human diseases including Alzheimer's disease, Parkinson's disease, and motor neuron disease. *α*_2_M is one of a small number of secreted proteins that are known to possess holdase-type chaperone activity, which is the ability to stabilise misfolded proteins and prevent their aberrant aggregation [16–20, 25]. The chaperone function of *α*_2_M has been demonstrated *in vitro* using a broad range of misfolded clients including denatured globular proteins and aggregation prone, intrinsically disordered substrates (e.g., A*β* peptide and Parkinson's disease-associated alpha-synuclein). Furthermore, it has been shown that *α*_2_M preferentially binds several plasma proteins *in situ* following experimentally-induced shear stress which causes plasma protein aggregation [18, 19]. The likely fate for complexes formed between native *α*_2_M and misfolded proteins is clearance via LRP1 following interaction with a protease [16, 22, 25–27] (Figure 1(a)). However, protease-transformed *α*_2_M can also inhibit A*β* aggregation via degrading the peptide because trapped proteases remain active following covalent binding to *α*_2_M [18, 19]. The neuroprotective activity of *α*_2_M against the toxicity induced by misfolded proteins has been demonstrated using several *in vitro* models [17, 25, 27, 28] and has also been demonstrated in rats directly injected with toxic A*β* oligomers [29]. Taken together, the results of these studies support the conclusion that the functions of *α*_2_M are broadly important to extracellular proteostasis.

## 2. *α*_2_M and Neurodegenerative Diseases

Interest in the role of *α*_2_M in Alzheimer's disease spans several decades. In part, this stems from early reports that polymorphisms in *α*_2_M are associated with increased risk of Alzheimer's disease in some populations [30–36]. However, opposing results have also been presented [37, 38], and more recent genome-wide association studies have not found any association [39]. It has recently been reported that serum *α*_2_M is elevated in men with preclinical Alzheimer's disease, which potentially represents a general response to neuronal injury [40]. The significance of elevated levels of *α*_2_M is hard to determine, because aside from influencing A*β* aggregation and clearance, there are many other relevant biological processes that *α*_2_M potentially influences. For example, apolipoprotein E (ApoE) is an endogenous ligand of *α*_2_M in blood plasma, and the binding of *α*_2_M to the *ε*4 isoform (the strongest known genetic risk factor for Alzheimer's disease) is much less compared to the binding of *α*_2_M to the *ε*2 and *ε*3 ApoE isoforms [15]. The functional importance of this interaction has yet to be solved.

There is strong evidence that native *α*_2_M can inhibit the aggregation and toxicity of A*β* peptide (the major constituent of extracellular plaques in Alzheimer's disease). Furthermore, the widely documented ability of *α*_2_M to facilitate the clearance of the A*β* peptide is central to its neuroprotective action [17, 25, 27–29]. *α*_2_M is found colocalised with the A*β* peptide in the brain in Alzheimer's disease [41, 42], which supports the idea that the LRP1-mediated clearance of *α*_2_M-A*β* complexes is impaired or overwhelmed. Similar to *α*_2_M, there are conflicting reports regarding an association between polymorphisms in LRP1 and the risk of Alzheimer's disease (reviewed in [43]). Given that the accumulation of the A*β* peptide in the brain in Alzheimer's disease appears to be the result of a defect in clearance, rather than elevated production of the peptide [44], it is important to understand the contribution of *α*_2_M to the clearance of the A*β* peptide in greater detail.

Roles for *α*_2_M in preventing or promoting neurodegeneration independent of Alzheimer's disease are less clear. Nevertheless, *α*_2_M is reported to bind to a broad range of misfolded proteins including the infectious prion protein that is responsible for transmissible spongiform encephalopathies [45] and *α*-synuclein, the major constituent of misfolded protein deposits in Parkinson's disease [17]. In the case of the prion protein, it has been reported that binding to *α*_2_M *in vitro* facilitates the conformational change in the prion protein that is responsible for its infectious characteristics [45]. On the other hand, similar to the protective effect of *α*_2_M on A*β* toxicity, the binding of *α*_2_M to *α*-synuclein is cytoprotective [17]. *α*_2_M also potentially inhibits neurodegeneration by influencing the activity of neurotrophins such as NGF and pro-NGF or by inhibiting the activity of neurotrophin receptors directly [12, 23, 24]. The latter could have relevance in a range of neurodegenerative diseases including Alzheimer's disease, Parkinson's disease, and Huntington's disease in which aberrant neurotrophin signalling is implicated [46]. Moreover, the ability of *α*_2_M to bind to proinflammatory mediators such as TNF-*α*, IL-6, and IL-1*β* [47–49] supports the idea that *α*_2_M has generalised importance in controlling inflammatory processes including in the central nervous system.

## 3. Hypochlorite, a Novel Regulator of *α*_2_M Functions

Hypochlorite (OCl^−^) is a powerful oxidant that is produced by the action of the enzyme myeloperoxidase during inflammation. Myeloperoxidase is not detected in the brains of healthy individuals; however, in neuroinflammatory disorders, myeloperoxidase is generated by activated microglia and astrocytes [50–54]. Infiltrating monocytes/macrophages and neutrophils can also contribute to myeloperoxidase production in the brain [50, 55]. Although the reasons for this are unclear, myeloperoxidase-immunoreactivity is also detected in neurons in Alzheimer's disease [50, 51]. Interestingly, in a mouse model of Parkinson's disease, ablation of the myeloperoxidase gene is protective, which supports the conclusion that myeloperoxidase is a major contributor to the oxidative damage generated by pathological neuroinflammatory processes [56].

Hypochlorite production is primarily considered important for defence against invading microbes [57]. The effectiveness of hypochlorite as a microbicidal agent is linked to the potency with which hypochlorite damages proteins, inducing their misfolding [58, 59]. Given that reaction with hypochlorite is not specific to molecules of microbial origin, the generation of hypochlorite is associated with collateral damage to the host organism. As a result of aberrant inflammatory activity, hypochlorite-modified proteins accumulate in a large number of pathologies including Alzheimer's disease [51], atherosclerosis [60], kidney disease [61], rheumatoid arthritis [52] and in experimental animal models of Parkinson's disease [56] and multiple sclerosis [62]. Hypochlorite-induced modification can directly cause proteins to adopt immunostimulatory and cytotoxic properties. For example, hypochlorite-induced modification of apolipoprotein B-100, the major protein component of low-density lipoprotein particles, promotes macrophage foam cell formation and triggers platelet aggregation [63]. Additionally, hypochlorite-modified albumin is known to promote proinflammatory signalling [64], endothelial cell dysfunction [65], and apoptosis [66].

**Figure 2:**
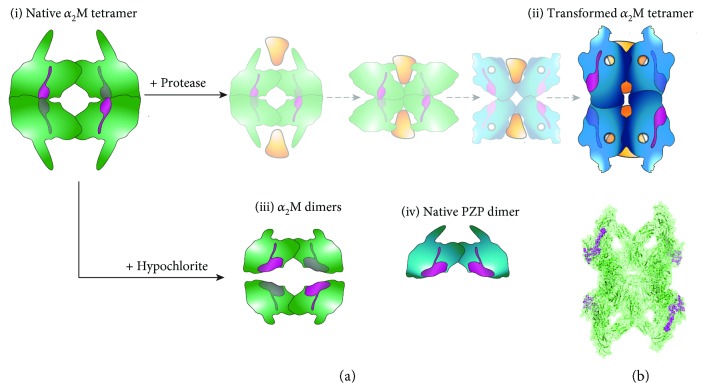
Theoretical model showing the binding sites for monomeric A*β* on native *α*_2_M and PZP. (a) The binding sites for monomeric A*β* (magenta; centred at amino acids 1314–1365 according to [21]) are normally concealed at the noncovalent interface of the (i) native *α*_2_M tetramer. (ii) Binding to proteases (yellow triangles) results in the partial opening of the noncovalent interface between *α*_2_M dimers and exposes the binding sites for monomeric A*β* on each subunit of transformed *α*_2_M. (iii) The binding sites for monomeric A*β* are also exposed by hypochlorite-induced dissociation of the native *α*_2_M tetramer into dimers. (iv) Native PZP (a disulfide-linked dimer) shares 82.7% sequence identity with *α*_2_M in the A*β* binding region (magenta). The dimeric quaternary structure of native PZP results in surface exposure of the binding sites for monomeric A*β*. Although the binding sites for other misfolded proteins are not known, intuitively, they are also located at the normally buried hydrophobic interface of noncovalently associated *α*_2_M dimers. (b) Image of the crystal structure of the transformed *α*_2_M tetramer from PBD 4ACQ [3] with the binding sites for monomeric A*β* shown in magenta, which is comparable to the model shown in (a (ii)). The crystal structures of native *α*_2_M or hypochlorite-modified *α*_2_M dimers have not been solved.

It is well-known that antioxidants are the first line of defence that protects the host from excessive oxidative damage during inflammation. However, evidence has emerged that supports the conclusion that specialised hypochlorite-inducible systems are also important. Around a decade ago, it was demonstrated that the activity of the bacterial chaperone Hsp33 is directly enhanced following reaction with hypochlorite and the chaperone activity of hypochlorite-modified Hsp33 protects bacteria from hypochlorite-induced death [59]. More recently, it has been demonstrated that reaction with hypochlorite induces the dissociation of the native *α*_2_M tetramer into dimers that have dramatically enhanced chaperone activity compared to the native *α*_2_M tetramer [25] (Figure 1(b)). The mechanism responsible for the enhanced chaperone activity of hypochlorite-modified *α*_2_M dimers involves the exposure of the normally buried hydrophobic surfaces that are situated at the interface of noncovalently-associated dimers in the native *α*_2_M tetramer [25] (Figure 2). It has been reported that methionine oxidation is largely responsible for the hypochlorite-induced dissociation of *α*_2_M into dimers [67]; however, aromatic amino acids are also modified by physiologically relevant levels of hypochlorite [25, 68, 69]. The results of biophysical analyses show that physiologically-relevant levels of hypochlorite also alter the secondary structure of *α*_2_M subunits [25, 68]. Precisely how hypochlorite-induced modification of the secondary structure of *α*_2_M influences its functions is not known.

During inflammation, extracellular protease activity and the generation of hypochlorite are both elevated; therefore, it is plausible that protease-transformed *α*_2_M and hypochlorite-induced *α*_2_M dimers are concomitantly generated *in vivo*. Hypochlorite-induced modification of native *α*_2_M exposes its LRP1 binding sites ([25, 70]); therefore, during inflammation, *α*_2_M and its cargoes are potentially cleared via two distinct mechanisms involving LRP1 (Figure 1(a): protease-transformed *α*_2_M and Figure 1(b): hypochlorite-induced *α*_2_M dimers). The dissociation constant for the binding of hypochlorite-modified *α*_2_M to LRP1 is reportedly ~0.7 nM [70] compared to 40 pM—2 nM for the transformed *α*_2_M [71]. Unlike native *α*_2_M, reaction with hypochlorite does not induce transformed *α*_2_M (generated using methylamine) to dissociate into dimers, and the resultant hypochlorite-induced damage reduces the binding of transformed *α*_2_M to LRP1 [70]. Therefore, during inflammation, the generation of hypochlorite potentially enhances the delivery of hypochlorite-modified *α*_2_M dimers that are generated from the native *α*_2_M tetramer to LRP1, while impeding the delivery of transformed *α*_2_M to the same receptor.

Although the chaperone activity of native *α*_2_M is enhanced following hypochlorite-induced modification, similar levels of hypochlorite-induced modification abolish the protease trapping function of *α*_2_M [72, 73]. Collectively, the evidence suggests that reaction with hypochlorite is a rapid switch that regulates the activities of *α*_2_M during inflammation. Supporting this idea, it has been reported that hypochlorite-induced modification of *α*_2_M also regulates its binding to cytokines and growth factors in a manner that increases its binding to TNF-*α*, IL-2, and IL-6 (involving preferential binding to hypochlorite-induced *α*_2_M dimers) and decreases its binding to *β*-NGF, PDGF-BB, TGF-*β*1, and TGF-*β*2 *in vitro* [74] (Figure 1(b)). Furthermore, hypochlorite-induced dissociation of *α*_2_M enhances its cytoprotective effect against TNF-*α in vitro* [74]. Interestingly, it has been reported that the complement system, which includes several proteins that are closely related to *α*_2_M, is also activated by reaction with hypochlorite [75, 76]. Therefore, it is tempting to speculate that hypochlorite-induced regulation is a characteristic that is shared by this family of proteins.

Studies of the hypochlorite-induced regulation of *α*_2_M are currently limited to *in vitro* systems; however, using the specific marker for reaction with hypochlorite 3-chlorotyrosine, it has been shown that *α*_2_M is modified by hypochlorite in synovial fluid from inflamed joints [69]. Moreover, considering that hypochlorite levels are predicted to reach the low millimolar range in tissues during inflammation [77], it is plausible that hypochlorite-modified *α*_2_M dimers are generated in biological fluids during inflammation. Of the studies reporting an association between mutation in *α*_2_M and risk of Alzheimer's disease, one study has reported that there is a synergistic effect between polymorphisms in *α*_2_M and myeloperoxidase and an increased risk of Alzheimer's disease [36]. The results of the latter study support the idea that the functions of these two proteins might interrelate in a way that is important to neurodegeneration. It is not currently known if any of the other identified extracellular chaperones (e.g., clusterin and haptoglobin) might also have their activities regulated by hypochlorite-induced modification, but this is an area worthy of future investigation.

## 4. PZP, a Dimeric *α*_2_M-like Molecule

The major structural modification induced by reaction with hypochlorite that is responsible for functionally controlling *α*_2_M is the dissociation of the native *α*_2_M tetramer into dimers. Strikingly, many mammals are capable of generating large amounts of a dimeric *α*_2_M-like protein known as pregnancy zone protein (PZP). In humans, *α*_2_M and PZP share very high sequence homology in all domains (71% amino acid identity), with the exception of the bait region [4, 78]. As a result, the ability of PZP to inhibit proteases is much more restricted compared to that of *α*_2_M. Few *in vitro* studies have focused on characterising the functions of PZP; however, it has been proposed that PZP contributes to regulating glycodelin-A (a paracrine mediator in early pregnancy) and TGF-*β*2 (important for embryonic development) [12, 79–81]. Consistent with this idea, PZP is usually lowly abundant in biological fluids but is markedly upregulated in pregnancy [82]. On the other hand, glycodelin-A and TGF-*β*2 are also ligands for constitutively abundant *α*_2_M ([12, 79–81]); therefore, the precise importance of PZP as a modulator of these signalling pathways remains unclear. Similarly, several neurotrophins are shared ligands of PZP and *α*_2_M, but the precise biological importance of these interactions is not known [12]. Pregnancy-independent expression of PZP is widely reported in diseases such as Alzheimer's disease [83, 84], Parkinson's disease [85], rheumatoid arthritis [86], Behcet's syndrome [87], psoriasis [88, 89], Chagas disease [90], viral infection [91, 92], inflammatory bowel disease [93], and cancers [94, 95]. The latter observations support the idea that the upregulation of PZP could be a general stress response that is related to chronic inflammation. This limits the usefulness of PZP as a diagnostic marker; however, the results of studies of lymphoma and arthritis patients suggest that PZP levels are potentially useful for monitoring disease progression [95, 96].

The ability of native tetrameric *α*_2_M to inhibit A*β* aggregation is restricted to binding to soluble A*β* oligomers formed early during the aggregation pathway [20]. In contrast, transformed *α*_2_M and hypochlorite-modified *α*_2_M dimers bind to monomeric A*β* [21, 25], presumably via the hydrophobic binding site (centred at amino acids 1314–1365) identified by [21] (Figure 2). Intuitively, surface exposure of this site contributes to the efficiency with which hypochlorite-modified *α*_2_M dimers inhibit A*β* amyloid formation compared to native *α*_2_M [25]. Similarly, the results of recent studies show that PZP binds to the monomeric A*β* peptide and prevents the aggregation of the A*β* peptide much more efficiently than native *α*_2_M [97]. Whether or not PZP contributes to the clearance of the A*β* peptide *in vivo* is currently unknown; however, it has been demonstrated that PZP levels are elevated in women with presymptomatic Alzheimer's disease and PZP is found colocalised with microglia around A*β* plaques in the brain in Alzheimer's disease [83, 84]. Combined, these observations suggest that PZP is likely to participate in A*β* homeostasis. Whether or not the role of PZP overlaps with or is discrete from that of *α*_2_M remains to be determined.

## 5. Concluding Remarks

*α*_2_M is a remarkably multifunctional protein that can influence a broad range of biological processes. Direct injection of *α*_2_M into inflamed joints has been shown to have protective effects in a rodent model of osteoarthritis ([98]); however, the efficacy and safety of this as a human therapy is not yet known. An alternative *α*_2_M-based anti-inflammatory strategy involves the oral administration of proteases, which is proposed to increase levels of transformed *α*_2_M in blood plasma [99, 100]. This strategy is clearly limited by the poor bioavailability of orally administered proteases, but this problem could potentially be overcome by the identification of bioavailable small molecule modifiers of *α*_2_M function.

Growing evidence suggests that hypochlorite-induced dissociation of *α*_2_M into dimers is a rapid switch that enhances the ability of *α*_2_M to facilitate the clearance of disease-associated misfolded proteins and proinflammatory cytokines during inflammation. This is potentially a broadly important process that occurs in response to inflammation, including in neurodegenerative disorders in which neuroinflammation is known to be an early event that precedes other pathological changes (reviewed in [101]). A deeper understanding of the physiological relevance of hypochlorite-induced *α*_2_M dimers has the potential to shed much needed light on the participation of *α*_2_M in controlling inflammatory processes and extracellular protein homeostasis during neuroinflammation.

## References

[1] Garton M. J., Keir G., Lakshmi M. V., Thompson E. J. (1991). Age-related changes in cerebrospinal fluid protein concentrations. *Journal of the Neurological Sciences*.

[2] Sottrup-Jensen L. (1989). Alpha-macroglobulins: structure, shape, and mechanism of proteinase complex formation. *The Journal of Biological Chemistry*.

[3] Marrero A., Duquerroy S., Trapani S. (2012). The Crystal Structure of Human *α*2-Macroglobulin Reveals a Unique Molecular Cage. *Angewandte Chemie (International Ed. in English)*.

[4] Sottrup-Jensen L., Sand O., Kristensen L., Fey G. H. (1989). The alpha-macroglobulin bait region. Sequence diversity and localization of cleavage sites for proteinases in five mammalian alpha-macroglobulins. *The Journal of Biological Chemistry*.

[5] Barrett A. J., Starkey P. M. (1973). The interaction of alpha 2-macroglobulin with proteinases. Characteristics and specificity of the reaction, and a hypothesis concerning its molecular mechanism. *The Biochemical Journal*.

[6] Barrett A. J., Brown M. A., Sayers C. A. (1979). The electrophoretically ‘slow’ and ‘fast’ forms of the alpha 2-macroglobulin molecule. *The Biochemical Journal*.

[7] Ashcom J. D., Tiller S. E., Dickerson K., Cravens J. L., Argraves W. S., Strickland D. K. (1990). The human alpha 2-macroglobulin receptor: identification of a 420-kD cell surface glycoprotein specific for the activated conformation of alpha 2-macroglobulin. *The Journal of Cell Biology*.

[8] Kristensen T., Moestrup S. K., Gliemann J., Bendtsen L., Sand O., Sottrup-Jensen L. (1990). Evidence that the newly cloned low-density-lipoprotein receptor related protein (LRP) is the alpha 2-macroglobulin receptor. *FEBS Letters*.

[9] Gonias S. L., Carmichael A., Mettenburg J. M., Roadcap D. W., Irvin W. P., Webb D. J. (2000). Identical or overlapping sequences in the primary structure of human alpha(2)-macroglobulin are responsible for the binding of nerve growth factor-beta, platelet-derived growth factor-BB, and transforming growth factor-beta. *The Journal of Biological Chemistry*.

[10] LaMarre J., Wollenberg G. K., Gonias S. L., Hayes M. A. (1991). Cytokine binding and clearance properties of proteinase-activated alpha 2-macroglobulins. *Laboratory Investigation*.

[11] Barcelona P. F., Saragovi H. U. (2015). A pro-nerve growth factor (proNGF) and NGF binding protein, *α*2-macroglobulin, differentially regulates p75 and TrkA receptors and is relevant to neurodegeneration ex vivo and in vivo. *Molecular and Cellular Biology*.

[12] Skornicka E. L., Shi X., Koo P. H. (2002). Comparative binding of biotinylated neurotrophins to alpha(2)-macroglobulin family of proteins: relationship between cytokine-binding and neuro-modulatory activities of the macroglobulins. *Journal of Neuroscience Research*.

[13] Westwood M., Aplin J. D., Collinge I. A., Gill A., White A., Gibson J. M. (2001). Alpha 2-macroglobulin: a new component in the insulin-like growth factor/insulin-like growth factor binding protein-1 axis. *The Journal of Biological Chemistry*.

[14] Wolf B. B., Gonias S. L. (1994). Neurotrophin binding to human alpha 2-macroglobulin under apparent equilibrium conditions. *Biochemistry*.

[15] Krimbou L., Tremblay M., Davignon J., Cohn J. S. (1998). Association of apolipoprotein E with alpha2-macroglobulin in human plasma. *Journal of Lipid Research*.

[16] French K., Yerbury J. J., Wilson M. R. (2008). Protease activation of alpha2-macroglobulin modulates a chaperone-like action with broad specificity. *Biochemistry*.

[17] Whiten D. R., Cox D., Horrocks M. H. (2018). Single-Molecule Characterization of the Interactions between Extracellular Chaperones and Toxic *α*-Synuclein Oligomers. *Cell Reports*.

[18] Wyatt A. R., Constantinescu P., Ecroyd H. (2013). Protease-activated alpha-2-macroglobulin can inhibit amyloid formation via two distinct mechanisms. *FEBS Letters*.

[19] Wyatt A. R., Zammit N. W., Wilson M. R. (2013). Acute phase proteins are major clients for the chaperone action of *α*2-macroglobulin in human plasma. *Cell Stress & Chaperones*.

[20] Yerbury J. J., Kumita J. R., Meehan S., Dobson C. M., Wilson M. R. (2009). Alpha2-macroglobulin and haptoglobin suppress amyloid formation by interacting with prefibrillar protein species. *The Journal of Biological Chemistry*.

[21] Mettenburg J. M., Webb D. J., Gonias S. L. (2002). Distinct binding sites in the structure of alpha 2-macroglobulin mediate the interaction with beta-amyloid peptide and growth factors. *The Journal of Biological Chemistry*.

[22] Narita M., Holtzman D. M., Schwartz A. L., Bu G. (2002). *α*2-Macroglobulin complexes with and mediates the endocytosis of *β*-amyloid peptide via cell surface low-density lipoprotein receptor-related protein. *Journal of Neurochemistry*.

[23] Koo P. H., Qiu W. S. (1994). Monoamine-activated alpha 2-macroglobulin binds trk receptor and inhibits nerve growth factor-stimulated trk phosphorylation and signal transduction. *The Journal of Biological Chemistry*.

[24] Liebl D. J., Koo P. H. (1993). Serotonin-activated alpha 2-macroglobulin inhibits neurite outgrowth and survival of embryonic sensory and cerebral cortical neurons. *Journal of Neuroscience Research*.

[25] Wyatt A. R., Kumita J. R., Mifsud R. W., Gooden C. A., Wilson M. R., Dobson C. M. (2014). Hypochlorite-induced structural modifications enhance the chaperone activity of human 2-macroglobulin. *Proceedings of the National Academy of Sciences of the United States of America*.

[26] Qiu Z., Strickland D. K., Hyman B. T., Rebeck G. W. (1999). Alpha2-macroglobulin enhances the clearance of endogenous soluble beta-amyloid peptide via low-density lipoprotein receptor-related protein in cortical neurons. *Journal of Neurochemistry*.

[27] Yerbury J. J., Wilson M. R. (2010). Extracellular chaperones modulate the effects of Alzheimer’s patient cerebrospinal fluid on Abeta(1-42) toxicity and uptake. *Cell Stress & Chaperones*.

[28] Fabrizi C., Businaro R., Lauro G. M., Fumagalli L. (2001). Role of alpha2-macroglobulin in regulating amyloid beta-protein neurotoxicity: protective or detrimental factor?. *Journal of Neurochemistry*.

[29] Cascella R., Conti S., Tatini F. (2013). Extracellular chaperones prevent A*β*42-induced toxicity in rat brains. *Biochimica et Biophysica Acta*.

[30] Alvarez V., Alvarez R., Lahoz C. H. (1999). Association between an alpha(2) macroglobulin DNA polymorphism and late-onset Alzheimer's disease. *Biochemical and Biophysical Research Communications*.

[31] Blacker D., Wilcox M. A., Laird N. M. (1998). Alpha-2 macroglobulin is genetically associated with Alzheimer disease. *Nature Genetics*.

[32] Liao A., Nitsch R. M., Greenberg S. M. (1998). Genetic association of an alpha2-macroglobulin (Val1000lle) polymorphism and Alzheimer’s disease. *Human Molecular Genetics*.

[33] Mariani E., Seripa D., Ingegni T. (2006). Interaction of CTSD and A2M polymorphisms in the risk for Alzheimer’s disease. *Journal of the Neurological Sciences*.

[34] Saunders A. J., Bertram L., Mullin K. (2003). Genetic association of Alzheimer’s disease with multiple polymorphisms in alpha-2-macroglobulin. *Human Molecular Genetics*.

[35] Xu X., Wang Y., Wang L. (2013). Meta-analyses of 8 polymorphisms associated with the risk of the Alzheimer’s disease. *PLoS One*.

[36] Zappia M., Manna I., Serra P. (2004). Increased risk for Alzheimer disease with the interaction of MPO and A2M polymorphisms. *Archives of Neurology*.

[37] Chen L., Baum L., Ng H. K. (1999). Apolipoprotein E promoter and *α*2-macroglobulin polymorphisms are not genetically associated with Chinese late onset Alzheimer’s disease. *Neuroscience Letters*.

[38] Wavrant-DeVrièze F., Rudrasingham V., Lambert J. C. (1999). No association between the alpha-2 macroglobulin I1000V polymorphism and Alzheimer’s disease. *Neuroscience Letters*.

[39] Shen L., Jia J. (2016). An overview of genome-wide association studies in Alzheimer’s disease. *Neuroscience Bulletin*.

[40] Varma V. R., Predictors of Cognitive Decline Among Normal Individuals (BIOCARD) and the Alzheimer’s Disease Neuroimaging Initiative (ADNI) studies, Varma S. (2017). Alpha-2 macroglobulin in Alzheimer’s disease: a marker of neuronal injury through the RCAN1 pathway. *Molecular Psychiatry*.

[41] Thal D. R., Schober R., Birkenmeier G. (1997). The subunits of alpha2-macroglobulin receptor/low density lipoprotein receptor-related protein, native and transformed alpha2-macroglobulin and interleukin 6 in Alzheimer’s disease. *Brain Research*.

[42] Van Gool D., de Strooper B., Van Leuven F., Triau E., Dom R. (1993). *α*2-macroglobulin expression in neuritic-type plaques in patients with Alzheimer's disease. *Neurobiology of Aging*.

[43] Shinohara M., Tachibana M., Kanekiyo T., Bu G. (2017). Role of LRP1 in the pathogenesis of Alzheimer’s disease: evidence from clinical and preclinical studies. *Journal of Lipid Research*.

[44] Deane R., Bell R., Sagare A., Zlokovic B. (2009). Clearance of amyloid-beta peptide across the blood-brain barrier: implication for therapies in Alzheimer’s disease. *CNS & Neurological Disorders Drug Targets*.

[45] Adler V., Davidowitz E., Tamburi P., Rojas P., Grossman A. (2007). *α*2-Macroglobulin is a potential facilitator of prion protein transformation. *Amyloid*.

[46] Meldolesi J. (2017). Neurotrophin receptors in the pathogenesis, diagnosis and therapy of neurodegenerative diseases. *Pharmacological Research*.

[47] Borth W., Urbanski A., Prohaska R., Susanj M., Luger T. A. (1990). Binding of recombinant interleukin-1 beta to the third complement component and alpha 2-macroglobulin after activation of serum by immune complexes. *Blood*.

[48] Matsuda T., Hirano T., Nagasawa S., Kishimoto T. (1989). Identification of alpha 2-macroglobulin as a carrier protein for IL-6. *The Journal of Immunology*.

[49] Wollenberg G. K., LaMarre J., Rosendal S., Gonias S. L., Hayes M. A. (1991). Binding of tumor necrosis factor alpha to activated forms of human plasma alpha 2 macroglobulin. *The American Journal of Pathology*.

[50] Gellhaar S., Sunnemark D., Eriksson H., Olson L., Galter D. (2017). Myeloperoxidase-immunoreactive cells are significantly increased in brain areas affected by neurodegeneration in Parkinson’s and Alzheimer’s disease. *Cell and Tissue Research*.

[51] Green P. S., Mendez A. J., Jacob J. S. (2004). Neuronal expression of myeloperoxidase is increased in Alzheimer’s disease. *Journal of Neurochemistry*.

[52] Stamp L. K., Khalilova I., Tarr J. M. (2012). Myeloperoxidase and oxidative stress in rheumatoid arthritis. *Rheumatology*.

[53] Lefkowitz D. L., Lefkowitz S. S. (2008). Microglia and myeloperoxidase: a deadly partnership in neurodegenerative disease. *Free Radical Biology & Medicine*.

[54] Maki R. A., Tyurin V. A., Lyon R. C. (2009). Aberrant expression of myeloperoxidase in astrocytes promotes phospholipid oxidation and memory deficits in a mouse model of Alzheimer disease. *The Journal of Biological Chemistry*.

[55] Matsuo Y., Onodera H., Shiga Y. (1994). Correlation between myeloperoxidase-quantified neutrophil accumulation and ischemic brain injury in the rat. Effects of neutrophil depletion. *Stroke*.

[56] Choi D. K., Pennathur S., Perier C. (2005). Ablation of the inflammatory enzyme myeloperoxidase mitigates features of Parkinson’s disease in mice. *The Journal of Neuroscience*.

[57] Lanza F. (1998). Clinical manifestation of myeloperoxidase deficiency. *Journal of Molecular Medicine (Berlin, Germany)*.

[58] Pattison D. I., Davies M. J. (2001). Absolute rate constants for the reaction of hypochlorous acid with protein side chains and peptide bonds. *Chemical Research in Toxicology*.

[59] Winter J., Ilbert M., Graf P. C. F., Özcelik D., Jakob U. (2008). Bleach activates a redox-regulated chaperone by oxidative protein unfolding. *Cell*.

[60] Hazell L. J., van den Berg J. J. M., Stocker R. (1994). Oxidation of low-density lipoprotein by hypochlorite causes aggregation that is mediated by modification of lysine residues rather than lipid oxidation. *The Biochemical Journal*.

[61] Malle E., Woenckhaus C., Waeg G., Esterbauer H., Gröne E. F., Gröne H. J. (1997). Immunological evidence for hypochlorite-modified proteins in human kidney. *The American Journal of Pathology*.

[62] Yu G., Zheng S., Zhang H. (2018). Inhibition of myeloperoxidase by N-acetyl lysyltyrosylcysteine amide reduces experimental autoimmune encephalomyelitis-induced injury and promotes oligodendrocyte regeneration and neurogenesis in a murine model of progressive multiple sclerosis. *Neuroreport*.

[63] Volf I., Roth A., Cooper J., Moeslinger T., Koller E. (2000). Hypochlorite modified LDL are a stronger agonist for platelets than copper oxidized LDL. *FEBS Letters*.

[64] Marsche G., Semlitsch M., Hammer A. (2007). Hypochlorite-modified albumin colocalizes with RAGE in the artery wall and promotes MCP-1 expression via the RAGE-Erk1/2 MAP-kinase pathway. *The FASEB Journal*.

[65] Tang D. D., Niu H. X., Peng F. F. (2016). Hypochlorite-Modified Albumin Upregulates ICAM-1 Expressionviaa MAPK–NF-*κ*B Signaling Cascade: Protective Effects of Apocynin. *Oxidative Medicine and Cellular Longevity*.

[66] Li Zhou L., Hou F. F., Wang G. B. (2009). Accumulation of advanced oxidation protein products induces podocyte apoptosis and deletion through NADPH-dependent mechanisms. *Kidney International*.

[67] Reddy V. Y., Desorchers P. E., Pizzo S. V. (1994). Oxidative dissociation of human alpha 2-macroglobulin tetramers into dysfunctional dimers. *The Journal of Biological Chemistry*.

[68] Siddiqui T., Zia M. K., Ali S. S., Ahsan H., Khan F. H. (2018). Insight into the interactions of proteinase inhibitor- alpha-2-macroglobulin with hypochlorite. *International Journal of Biological Macromolecules*.

[69] Wu S. M., Pizzo S. V. (2001). Alpha(2)-macroglobulin from rheumatoid arthritis synovial fluid: functional analysis defines a role for oxidation in inflammation. *Archives of Biochemistry and Biophysics*.

[70] Wu S. M., Boyer C. M., Pizzo S. V. (1997). The binding of receptor-recognized alpha2-macroglobulin to the low density lipoprotein receptor-related protein and the alpha2M signaling receptor is decoupled by oxidation. *The Journal of Biological Chemistry*.

[71] Moestrup S. K., Gliemann J. (1991). Analysis of ligand recognition by the purified alpha 2-macroglobulin receptor (low density lipoprotein receptor-related protein) Evidence that high affinity of alpha 2-macroglobulin-proteinase complex is achieved by binding to adjacent receptors. *The Journal of Biological Chemistry*.

[72] Abbink J. J., Kamp A. M., Nieuwenhuys E. J., Nuijens J. H., Swaak A. J. G., Hack C. E. (1991). Predominant role of neutrophils in the inactivation of alpha 2-macroglobulin in arthritic joints. *Arthritis and Rheumatism*.

[73] Wu S. M., Pizzo S. V. (1999). Mechanism of hypochlorite-mediated inactivation of proteinase inhibition by alpha 2-macroglobulin. *Biochemistry*.

[74] Wu S. M., Patel D. D., Pizzo S. V. (1998). Oxidized *α*2-Macroglobulin (*α*2M) Differentially Regulates Receptor Binding by Cytokines/Growth Factors: Implications for Tissue Injury and Repair Mechanisms in Inflammation. *Journal of Immunology*.

[75] Shingu M., Nonaka S., Nishimukai H., Nobunaga M., Kitamura H., Tomo-Oka K. (1992). Activation of complement in normal serum by hydrogen peroxide and hydrogen peroxide-related oxygen radicals produced by activated neutrophils. *Clinical and Experimental Immunology*.

[76] Vogt W. (1996). Complement activation by myeloperoxidase products released from stimulated human polymorphonuclear leukocytes. *Immunobiology*.

[77] Weiss S. J. (1989). Tissue destruction by neutrophils. *The New England Journal of Medicine*.

[78] Devriendt K., van den Berghe H., Cassiman J. J., Marynen P. (1991). Primary structure of pregnancy zone protein. Molecular cloning of a full-length PZP cDNA clone by the polymerase chain reaction. *Biochimica et Biophysica Acta*.

[79] Chiabrando G. A., Sánchez M. C., Skornicka E. L., Koo P. H. (2002). Low-density lipoprotein receptor-related protein mediates in PC12 cell cultures the inhibition of nerve growth factor-promoted neurite outgrowth by pregnancy zone protein and alpha2-macroglobulin. *Journal of Neuroscience Research*.

[80] Philip A., Bostedt L., Stigbrand T., O'connor-McCourt M. D. (1994). Binding of transforming growth factor‐*β* (TGF‐*β*) to pregnancy zone protein (PZP). *European Journal of Biochemistry*.

[81] Skornicka E. L., Kiyatkina N., Weber M. C., Tykocinski M. L., Koo P. H. (2004). Pregnancy zone protein is a carrier and modulator of placental protein-14 in T-cell growth and cytokine production. *Cellular Immunology*.

[82] Ekelund L., Laurell C. . B. (1994). The pregnancy zone protein response during gestation: a metabolic challenge. *Scandinavian Journal of Clinical and Laboratory Investigation*.

[83] IJsselstijn L., Dekker L. J. M., Stingl C. (2011). Serum levels of pregnancy zone protein are elevated in presymptomatic Alzheimer’s disease. *Journal of Proteome Research*.

[84] Nijholt D. A. T., Ijsselstijn L., van der Weiden M. M. (2015). Pregnancy zone protein is increased in the Alzheimer’s disease brain and associates with senile plaques. *Journal of Alzheimer's Disease*.

[85] Henderson-Smith A., Corneveaux J. J., de Both M. (2016). Next-generation profiling to identify the molecular etiology of Parkinson dementia. *Neurology Genetics*.

[86] Horne C., Thomson A. W., Hunter C. B., Tunstall A. M., Towler C. M., Billingham M. E. (1979). Pregnancy-associated alpha 2-glycoprotein (alpha 2-PAG) and various acute phase reactants in rheumatoid arthritis and osteoarthritis. *Biomedicine*.

[87] Thomson A. W., Lehner T., Adinolfi M., Horne C. H. W. (1981). Pregnancy-associated alpha-2-glycoprotein in recurrent oral ulceration and Behçet’s syndrome. *International Archives of Allergy and Immunology*.

[88] Beckman L., Bergdahl K., Cedergren B. (1977). Increased serum levels of the pregnancy zone protein in psoriasis. *Acta Dermato-Venereologica*.

[89] Beckman L., Bergdahl K., Cedergren B. (1979). Association between Duffy blood groups and serum level of the pregnancy zone protein. *Human Heredity*.

[90] Ramos A. (2002). Trypanosoma cruzi: cruzipain and membrane-bound cysteine proteinase isoform(s) interacts with human alpha(2)-macroglobulin and pregnancy zone protein. *Experimental Parasitology*.

[91] Sarcione E. J., Biddle W. C. (2001). Elevated serum pregnancy zone protein levels in HIV-1-infected men. *AIDS*.

[92] Zarzur J. A., Aldao M., Sileoni S., Vides M. A. (1989). Serum pregnancy-associated alpha 2-glycoprotein levels in the evolution of hepatitis B virus infection. *Journal of Clinical Laboratory Analysis*.

[93] Viennois E., Baker M. T., Xiao B., Wang L., Laroui H., Merlin D. (2015). Longitudinal study of circulating protein biomarkers in inflammatory bowel disease. *Journal of Proteomics*.

[94] Stimson W. H. (1975). Variations in the level of a pregnancy-associated alpha-macroglobulin in patients with cancer. *Journal of Clinical Pathology*.

[95] Zalazar F. E., Chiabrando G. A., de Aldao N. A., Ojeda F., Vides M. A., Aldao M. A. J. (1992). Pregnancy-associated *α*2-glycoprotein in children with acute lymphocytic leukemia, Hodgkin’s disease and non-Hodgkin’s lymphomas. *Clinica Chimica Acta*.

[96] Unger A., Kay A., Griffin A. J., Panayi G. S. (1983). Disease activity and pregnancy associated alpha 2-glycoprotein in rheumatoid arthritis during pregnancy. *British Medical Journal (Clinical Research Ed.)*.

[97] Cater J. H., Kumita J. R., Zeineddine Abdallah R. (2019). Human pregnancy zone protein stabilizes misfolded proteins including preeclampsia- and Alzheimer’s-associated amyloid beta peptide. *Proceedings of the National Academy of Sciences*.

[98] Zhang Y., Wei X., Browning S., Scuderi G., Hanna L. S., Wei L. (2017). Targeted designed variants of alpha-2-macroglobulin (A2M) attenuate cartilage degeneration in a rat model of osteoarthritis induced by anterior cruciate ligament transection. *Arthritis Research & Therapy*.

[99] Leipner J., Saller R. (2000). Systemic enzyme therapy in oncology. *Drugs*.

[100] Lorkowski G. (2012). Gastrointestinal absorption and biological activities of serine and cysteine proteases of animal and plant origin: review on absorption of serine and cysteine proteases. *International Journal of Physiology, Pathophysiology and Pharmacology*.

[101] Heneka M. T., Carson M. J., Khoury J. E. (2015). Neuroinflammation in Alzheimer’s disease. *The Lancet Neurology*.

